# Correction: Xi et al. *Aerococcus viridans* Phage Lysin AVPL Had Lytic Activity against *Streptococcus suis* in a Mouse Bacteremia Model. *Int. J. Mol. Sci.* 2023, *24*, 16670

**DOI:** 10.3390/ijms27031296

**Published:** 2026-01-28

**Authors:** Hengyu Xi, Yao Fu, Chong Chen, Xin Feng, Wenyu Han, Jingmin Gu, Yalu Ji

**Affiliations:** 1 State Key Laboratory for Diagnosis and Treatment of Severe Zoonotic Infectious Diseases, Key Laboratory for Zoonosis Research of the Ministry of Education, Institute of Zoonosis, College of Veterinary Medicine, Jilin University, Changchun 130062, China; m15543135579@163.com (H.X.); fuyao0519@126.com (Y.F.); m13944830520@163.com (C.C.); hanwy@jlu.edu.cn (W.H.); jingmin0629@163.com (J.G.); 2 Jiangsu Co-Innovation Center for the Prevention and Control of Important Animal Infectious Diseases and Zoonoses, Yangzhou University, Yangzhou 225009, China

In the original publication [1], there was a mistake in Figure 5A as published. Specifically, in Figure 5A, the authors inadvertently pasted the histopathological pictures of the kidneys from the normal group to the AVPL group repeatedly, resulting in the misuse of the pictures. These duplications resulted from an oversight during the final figure assembly. The corrected Figure 5 appears below. The authors state that the scientific conclusions are unaffected. This correction was approved by the Academic Editor. The original publication has also been updated.

## Figures and Tables

**Figure 5:**
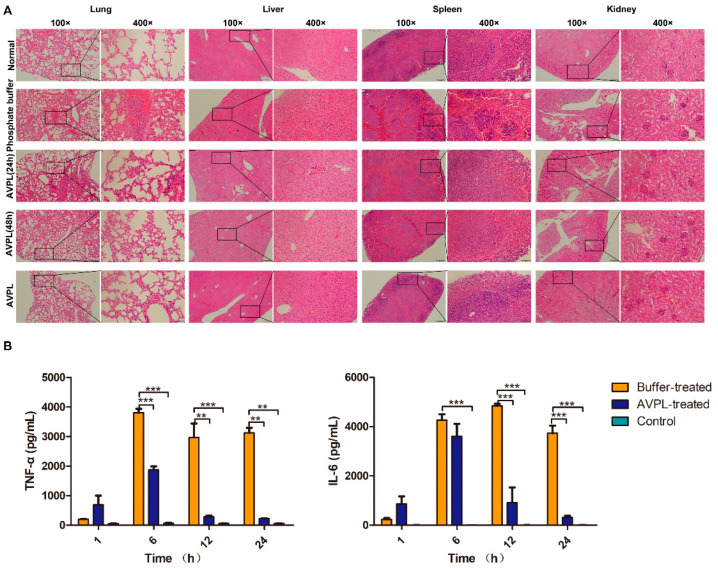
Pathological changes and cytokine levels. (**A**) Histopathology following *S. suis* infection. Lungs, liver, spleen, and kidneys from each group of mice were taken and stained with H&E (magnification, ×100, ×400) at 24 h and 48 h postinfection. A single dose of 600 µg/mouse AVPL was administered intraperitoneally for safety analysis. (**B**) The cytokine levels in mice were determined. IL-6 and TNF-α serum levels were measured in each group of mice at 1 h, 6 h, 12 h, and 24 h postinfection. Sera from healthy mice were used as controls. ** *p* < 0.01, *** *p* < 0.001. Data represent the mean ± SD (*n* = 3).

## References

[1] Xi H., Fu Y., Chen C., Feng X., Han W., Gu J., Ji Y. (2023). *Aerococcus viridans* Phage Lysin AVPL Had Lytic Activity against *Streptococcus suis* in a Mouse Bacteremia Model. Int. J. Mol. Sci..

