# Supplementary material for: Aerococcus viridans Phage Lysin AVPL Had Lytic Activity against Streptococcus suis in a Mouse Bacteremia Model
Source: Int J Mol Sci. 2023 Nov 23;24(23):16670. doi: 10.3390/ijms242316670 (PMC10706753; doi:10.3390/ijms242316670)
Supplement: Supplementary file 1 [file ijms-24-16670-s001.zip › ijms-2694791-supplementary.pdf]

Supplementary Materials

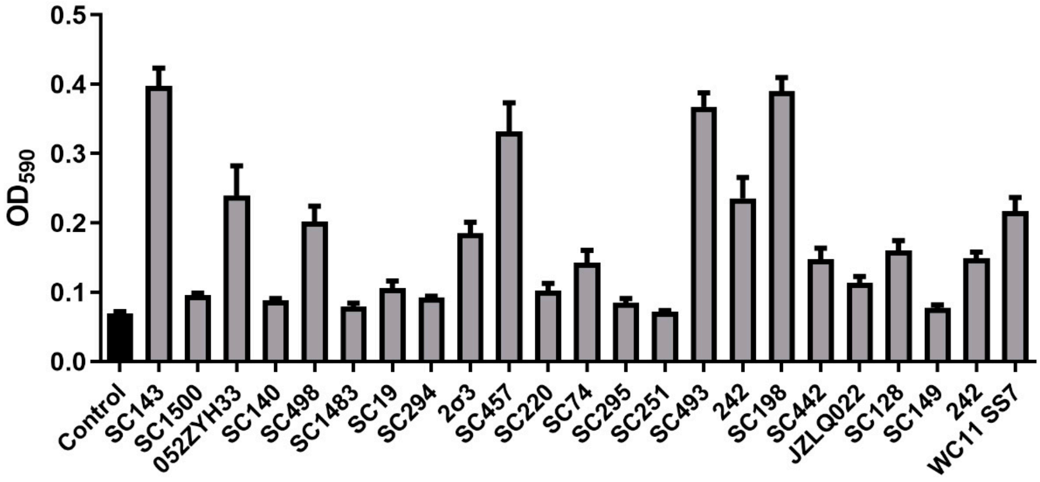

Figure. S1 Biofilm formation capacity of different *S. suis*. The strains were grown in 96-well microtiter dishes at 37°C for 48 h without agitation. The remaining cells were washed and stained with CV to determine OD_{590}. The above values represent the mean ±SD (n = 3).

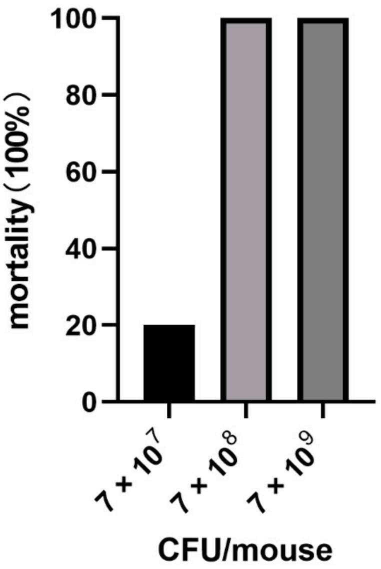

Figure S2 Mouse mortality determination. Mice were injected intraperitoneally with 100 μL of SC19 (7 × 10^7, 7 × 10^8, and 7 × 10^9 CFU/mouse, n = 10). Mortality was recorded over 7 days.

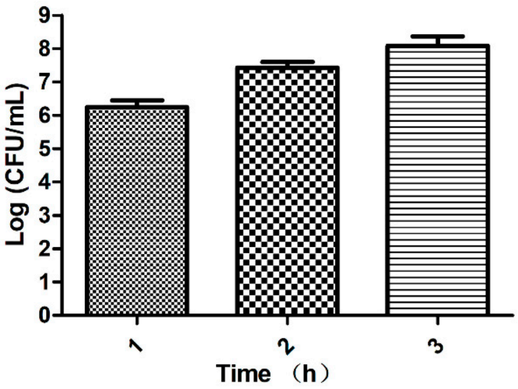

Figure S3 Bacterial burden in blood. Bacterial burden in blood at 1-3 h after inoculation of a lethal dose of SS2 SC19 was detected. Tail vein blood was collected from mice at 1, 2, and 3 h after the challenge and counted on BHI plates after multiplicative dilution. The values represent the mean ±SD (n = 3).

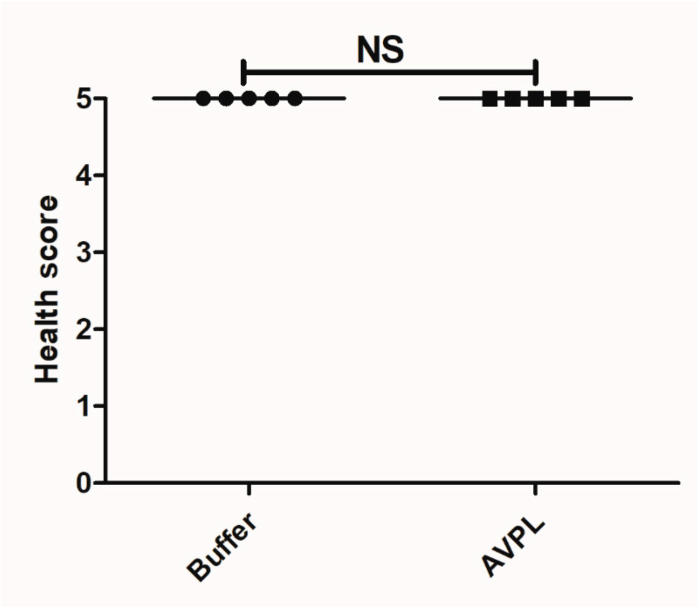

Figure S4. Health score. Two groups of mice (n = 5 per group) were intraperitoneally injected with 600 μg/mouse AVPL or phosphate buffer. The health status of the mice was scored on a scale of 0–5 at 7 d after treatment, with a score of 5 indicating normal health with no obvious symptoms, 4 indicating light disease (defined as reduced physical activity and fur wrinkling), 3 indicating moderate illness (defined as lethargy and hunchback), 2 indicating severe illness (defined as the above signs plus accumulation of exudate around partially closed eyes), 1 indicating moribund, and 0 indicating death. Each point indicates the health status of an individual mouse. NS, no significance.

Table S1. The strains used to detect the lytic activity of AVPL.

Organism	Strain	Spot	Source
S. suis	052ZYH33[2]	+	a
	JZLQ022[2]	+	a
	SC19[2]	+	a
	SC140[3]	+	b
	SC149[3]	+	b
	SC251[3]	+	b
	SC220[3]	+	b
	SC1483[3]	+	b
	SC294[3]	+	b
	SC295[3]	+	b
	SC1500[3]	+	b
	SC450[3]	+	b
	2θ3[7]	+	a
	242[7]	+	a
	WC11 SS7[7]	+	a
	JZLQ001[7]	+	a
	SC74[9]	+	b
	SC143[9]	+	b
	SC498[9]	+	b
	SC493[9]	+	b
	SC457[9]	+	b
	SC442[9]	+	b
	SC198[12]	+	b
	SC128[27]	+	b
	CPD16[2]	+	d
	CPD17[2]	+	d
	CPD28[2]	+	d
	CPD35[2]	+	d
	CPD40[2]	+	d
	CPD41[2]	+	d
S. pneumoniae	9894	-	a
	9488	-	a
S. agalactiae	B2200 215-3	-	a
S. dysgalactiae	0339	-	a
S. equi	seq 1	-	c
	Seq 2	-	c
	Seq 5	-	c
	Seq 6	-	c
	Seq 7	-	c
	Seq 8	-	c
	Seq 9	-	c
	Seq 10	-	c

	Seq 11	-	c
	Seq 12	-	c
	Seq 13	-	c
	Seq 14	-	c
	Seq 16	_	c
	Seq 17	_	c
	23ED	_	c
S. aureus	USA300	-	a
	N25	-	a
	JP2	-	a
	R6046	-	a
	SL1	-	a
	R238	-	a
	SW1	-	a
	R5886	-	a
	JP13	_	a
	R3784	-	a
	R6186	-	a
	N26	-	a
	R89	-	a
	JP14	-	a
	JP 5	-	a
	JP 1	-	a
	CVCC 2261	_	a
	N3	-	a
	R6199	-	a
	YB57	-	a
	R23	-	a
	R15	-	a
	W4727	-	a
	R6166	-	a
	JP 16	_	a
	SH1	-	a
	W4552	-	a
	JP 6	-	a
	JP 4	-	a
	JP 3	-	a
	J 2	-	a
	JP 7	-	a
	JP 10	_	a
	N 38	-	a
	JP 15	-	a
	W4661	-	a
	208	-	a

	W903	-	a
	R6016	-	a
	W3275	-	a
	JP 11	_	a
	R196	-	a
	R3790	-	a
	N4	-	a
	413	-	a
	WOOD 46	-	a
	JP 9	-	a
	ATCC 19685	-	a
	ATCC 26003	_	a
	ATCC 29213	-	a
	ATCC 49525	-	a
	ATCC 25923	-	a
A. baumannii	ABC 8	-	a
	ABC 27	-	a
	ABC 38	-	a
K. pneumoniae	Kpp 3	-	a
	Kpp 6	-	a
	Kpn5	-	a
S. enterica	ATCC 9842	-	a
S. typhimurium	O1E	-	a
E. faecalis	N 9	-	a
	Z 3	-	a
	Z 4	-	a
	ZC 12	-	a
	ZC 28	-	a
	ZJ 14	-	a
	ZC12	-	a
	ZNH 23	-	a
	ZNH 11	-	a
	GF 26	-	a
	FA 1	-	a
	FA 4	-	a
	FA 8	-	a
	002	-	a
E. faecium	SA 3	-	a
	SA 18	-	a
	SA 22	-	a
	V 02	-	a
	V 03	-	a
	V 09	-	a
	V 12	-	a

a, Stored in our lab; b, Provided by Professor Xiang-Dang Du at Henan Agricultural University; c, Provided by Professor Jian-bao Dong at Shandong Vocational Animal Science and Veterinary College; d, Provided by Professor Jinquan Li at Huazhong Agricultural University. The superscript numbers represent the serotypes of the different strains of *S. suis*, respectively.

Table S2. Determination of antibiotics susceptibility of *S. suis*.

Strain	MEM	P	CC	LEV	CTR	E	TE	FON	VA
052ZYH33	S	R	R	S	R	R	R	R	R
JZLQ022	S	I	R	S	R	I	I	S	R
SC19	S	R	S	S	R	I	R	S	S
CPD16	S	S	S	S	R	R	R	S	S
CPD17	S	S	I	S	R	I	R	S	S
CPD28	S	I	R	I	R	R	R	S	I
CPD35	S	S	R	S	S	R	R	S	S
CPD40	S	S	R	S	I	R	R	S	S
CPD41	S	R	R	S	R	R	R	S	S
SC140	S	I	R	S	R	R	R	I	I
SC149	S	I	R	S	R	R	I	S	S
SC251	S	I	R	I	I	I	R	I	S
SC220	S	S	R	S	S	I	R	S	S
SC1483	S	I	R	S	R	R	R	S	S
SC294	S	S	R	S	S	I	R	S	I
SC295	S	S	R	S	R	R	R	S	S
SC1500	S	S	R	I	S	R	R	R	S
SC450	S	S	R	S	S	R	R	S	I
2θ3	S	I	R	S	S	R	R	S	S
242	S	S	R	R	S	R	R	S	S
WC11 SS7	S	R	S	S	S	I	R	S	I
JZLQ001	S	S	R	S	S	I	R	S	I
SC74	S	S	R	S	R	R	R	S	S
SC143	S	I	I	S	S	I	R	I	S
SC498	S	R	R	I	S	R	R	S	S
SC493	S	I	R	S	R	R	R	S	I
SC457	S	S	R	S	S	I	R	S	S

SC442	S	S	R	S	R	I	R	S	I
SC198	S	I	R	R	I	I	R	S	S
SC128	S	S	R	R	S	R	R	S	S

MEM, Meropenem; P, Penicillin; CC, Clindamycin; LEV, Levofloxacin; CTR, Ceftriaxone; E, Erythromycin; TE, Tetracycline; FON, Florfenicol; VA, Vancomycin. S=Susceptible, I=Intermediate and R=Resistant.

Table S3. The primers used in this study.

Primers	Sequence (from 5' to 3')
AVPL-F	TACTTCCAATCCAATGCTATGGCAATTCCAAATAGTGCT
AVPL-R	TTATCCACTTCCAATGTTTAATCTTTTACTTCTTCAAC
EGFP-AVPL-C-F	GCGGCAGCCATATGCTCGAGATGGTGAGCAAGGGCGAG
EGFP-AVPL-C-R	AGGCTTACCTTTACCAGAACCCCCCTTGTACAGCT
Linker-C-F	TACAAGGGGGGTTCTGGTAAAGGTAAGCCTGATGGTATT
Linker-C-R	CTTTGTTAGCAGCCGGATCCTTAACCATGTCTTTGTAGGTAAGCA
EGFP-AVPL-B1-F	GCGGCAGCCATATGCTCGAGATGACTAAAGCTTACTATGATATTC
EGFP-AVPL-B1-F	CTCGCCCTTGCTCACAGAACCCCCGTTAGAGTTTGCT
Linker-B1-F	TCTAACGGGGGTTCTGTGAGCAAGGGCGAGGAG
Linker-B1-R	CTTTGTTAGCAGCCGGATCCCTACTTGTACAGCTCGTCCATG
EGFP-AVPL-B2-F	GCGGCAGCCATATGCTCGAGATGTTACAAAAATATGTAGGTAACG
EGFP-AVPL-B2-R	CTCGCCCTTGCTCACAGAACCCCCATCTTTTACTTCTTCAA
Linker-B2-F	AAAGATGGGGGTTCTGTGAGCAAGGGCGAGGAG
Linker-B2-R	CTTTGTTAGCAGCCGGATCCCTACTTGTACAGCTCGTCCATG
EGFP-AVPL-B1B2-F	GCGGCAGCCATATGCTCGAGATGACTAAAGCTTACTATGATATTC
EGFP-AVPL-B1B2-R	GCCCTTGCTCACCATAGAACCCCCTGGGGCACT
Linker-B1B2-F	GCCCCAGGGGGTTCTATGGTGAGCAAGGGCGAG
Linker-B1B2-R	CTTTGTTAGCAGCCGGATCCTTACTTGTACAGCTCGTCCATG

The restriction sites and homology arms are underlined.
